# Supplementary material for: Causal Relationship between Aging and Anorexia Nervosa: A White-Matter-Microstructure-Mediated Mendelian Randomization Analysis
Source: Biomedicines. 2024 Aug 16;12(8):1874. doi: 10.3390/biomedicines12081874 (PMC11351342; doi:10.3390/biomedicines12081874)
Supplement: Supplementary file 1 [file biomedicines-12-01874-s001.zip › Supplementary Figure/Figure S4/Singe SNP plot of MR analysis for TL to ubm-b-1860.pdf]

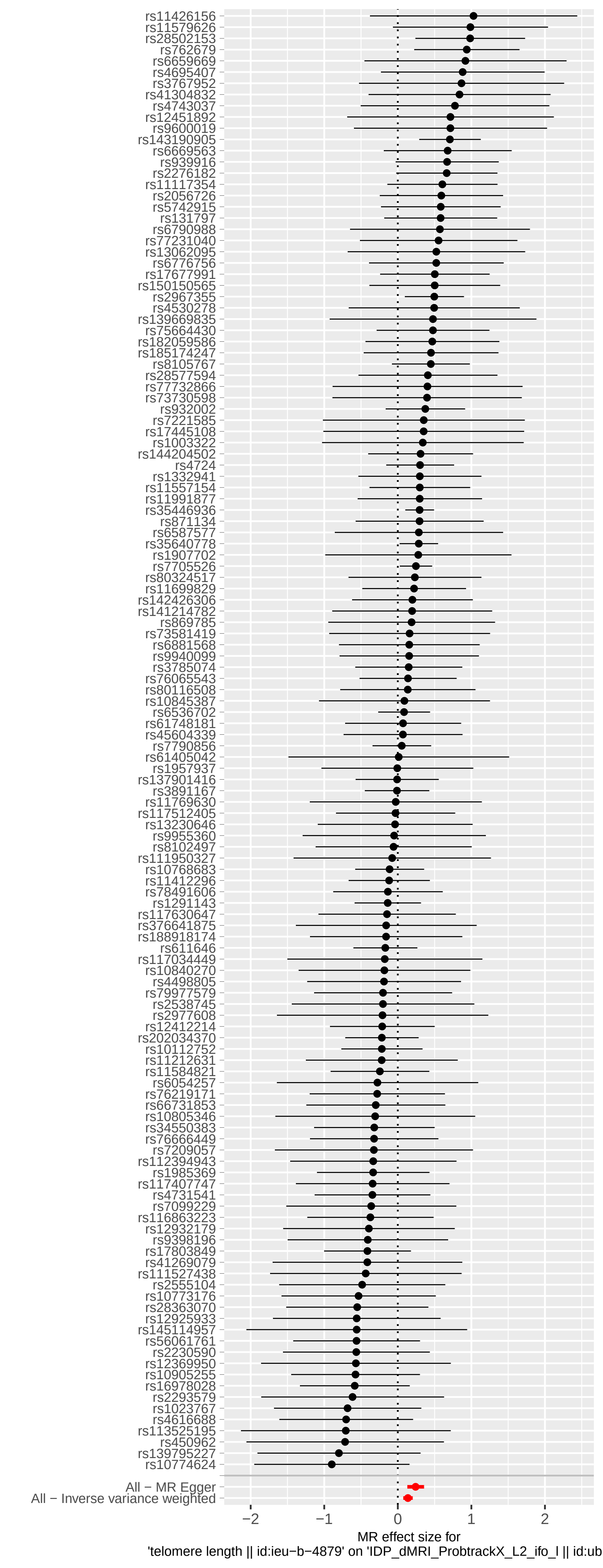
rs11426156
rs11579626
rs28502153
rs762679
rs6659669
rs4695407
rs3767952
rs41304832
rs4743037
rs12451892
rs9600019
rs143190905
rs6669563
rs939916
rs2276182
rs11117354
rs2056726
rs5742915
rs131797
rs6790988
rs77231040
rs13062095
rs6776756
rs17677991
rs150150565
rs2967355
rs4530278
rs139669835
rs75664430
rs182059586
rs185174247
rs8105767
rs28577594
rs77732866
rs73730598
rs932002
rs7221585
rs17445108
rs1003322
rs144204502
rs4724
rs1332941
rs11557154
rs11991877
rs35446936
rs871134
rs6587577
rs35640778
rs1907702
rs7705526
rs80324517
rs11699829
rs142426306
rs141214782
rs869785
rs73581419
rs6881568
rs9940099
rs3785074
rs76065543
rs80116508
rs10845387
rs6536702
rs61748181
rs45604339
rs7790856
rs61405042
rs1957937
rs137901416
rs3891167
rs11769630
rs117512405
rs13230646
rs9955360
rs8102497
rs111950327
rs10768683
rs11412296
rs78491606
rs1291143
rs117630647
rs376641875
rs188918174
rs611646
rs117034449
rs10840270
rs4498805
rs79977579
rs2538745
rs2977608
rs12412214
rs202034370
rs10112752
rs11212631
rs11584821
rs6054257
rs76219171
rs66731853
rs10805346
rs34550383
rs76666449
rs7209057
rs112394943
rs1985369
rs117407747
rs4731541
rs7099229
rs116863223
rs12932179
rs9398196
rs17803849
rs41269079
rs111527438
rs2555104
rs10773176
rs28363070
rs12925933
rs145114957
rs56061761
rs2230590
rs12369950
rs10905255
rs16978028
rs2293579
rs1023767
rs4616688
rs113525195
rs450962
rs139795227
rs10774624
All − MR Egger
All − Inverse variance weighted
−2
−1
0
1
2
MR effect size for
'telomere length || id:ieu−b−4879' on 'IDP_dMRI_ProbtrackX_L2_ifo_l || id:ub
